# Uncovering the Unexpected—True Lumen Compression by Subintimal Stent Causing Recurrent Acute Limb Ischemia for 15 Years: A Case Report

**DOI:** 10.1016/j.jscai.2026.105496

**Published:** 2026-07-16

**Authors:** Gordon Wong, Pedro S. Teixeira, Omar Sheikh, Mehdi H. Shishehbor

**Affiliations:** University Hospitals Harrington Heart & Vascular Institute, Case Western Reserve University School of Medicine, Cleveland, Ohio

**Keywords:** acute limb ischemia, case report, subintimal stent

## Abstract

The mechanism of stent failure in acute limb ischemia includes a broad spectrum of thrombotic and mechanical factors. We present a patient with recurring Rutherford Class 2A acute limb ischemia, who underwent peripheral angiography demonstrating severe compression of the left superficial femoral artery true lumen by an old subintimal stent. This compression—the cause of recurring limb ischemia events over 15 years—was treated with side-by-side stenting of the true lumen. Diagnosed by fluoroscopy and intravascular ultrasound, true lumen compression by a subintimal stent is a rare mechanical complication of peripheral angioplasty and requires true lumen stenting for definitive management.

## Introduction

Subintimal stenting for peripheral arterial disease commonly occurs in the treatment of long chronic total occlusions, particularly in the superficial femoral arteries. Occlusion of the false lumen stents can produce compression of the true lumen, leading to acute vessel closure and limb ischemia. When the distinction of true vs false lumen is missed, revascularization may fail to address the underlying cause of vessel closure. We present a case of a patient with recurrent limb ischemia due to subintimal stent compression of the true lumen.

## Patient information

A 69-year-old man with an extensive history of peripheral artery disease with recurring thromboses of the left superficial femoral artery stent (SFA) presented to the emergency room for progressive rest pain in his left foot that worsens with ambulation. His symptoms started 1 week before presentation. He has an extensive history of left lower extremity revascularization, including a distal left SFA stent placed 15 years prior and 2 occluded bypass grafts (left femoropopliteal and left profunda-to-anterior tibial [AT] artery). His most recent intervention recanalized an occluded left SFA and popliteal artery, with percutaneous transluminal angioplasty (PTA) and drug-eluting stent placement in the proximal SFA, and a bioresorbable scaffold implant in the left AT artery. He is on chronic dual antiplatelet therapy (DAPT) in addition to oral anticoagulation with warfarin. Other medical history includes type 2 diabetes mellitus, hypertension, and hyperlipidemia.

## Clinical findings

His vital signs were stable and within normal limits, and focused examination of the left foot was notable for dependent rubor with nonpalpable, faintly Dopplerable (monophasic) pulses in the dorsalis pedis (DP) and posterior tibial (PT) arteries. There was minimal swelling of the left calf and foot, with preserved motor function and minimal sensory changes.

## Diagnostic assessment

**Figure 1:**
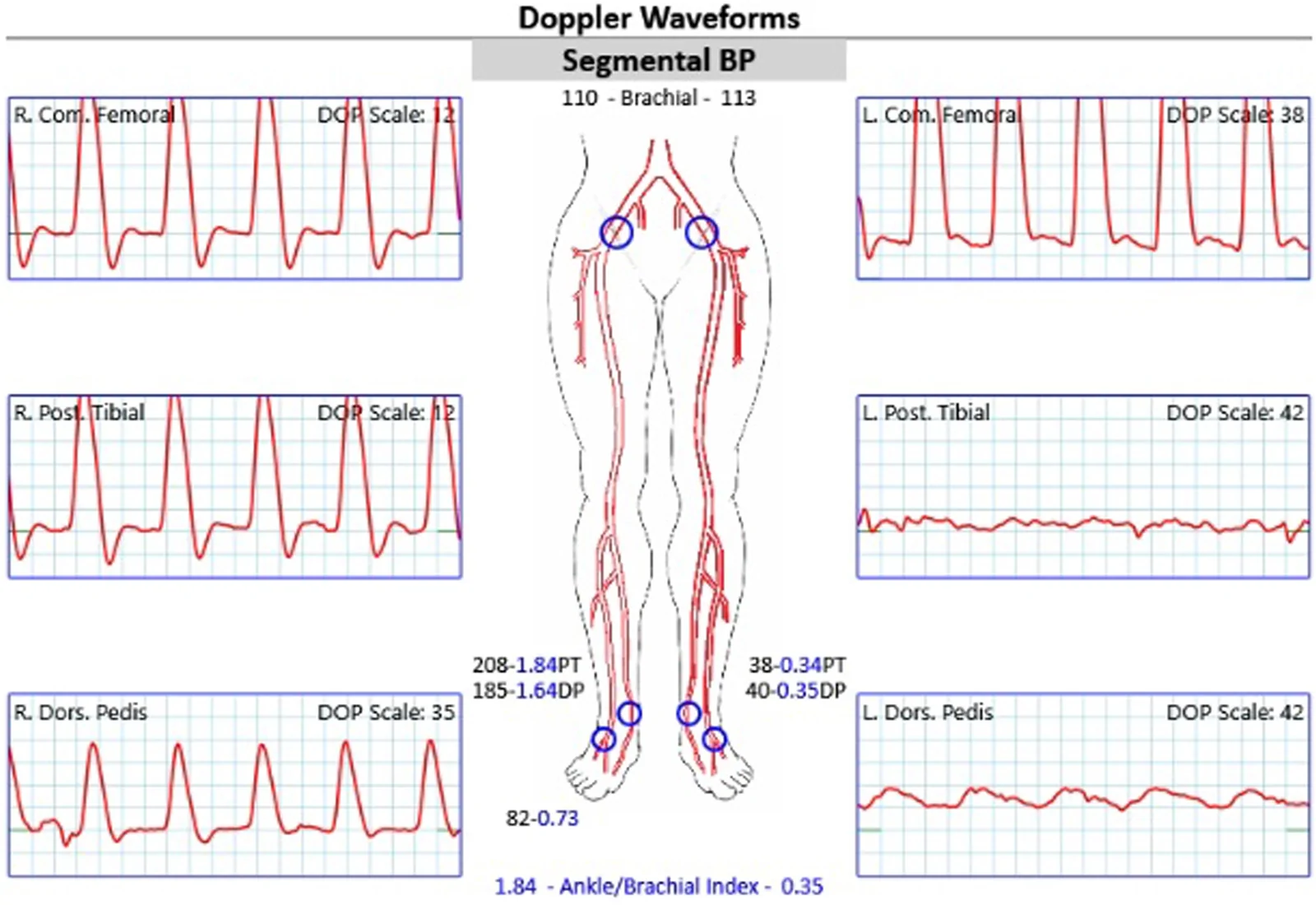
**Preintervention ABI showing low amplitude, monophasic waveforms of the left lower extremity.** ABI, ankle-brachial index.

**Figure 2:**
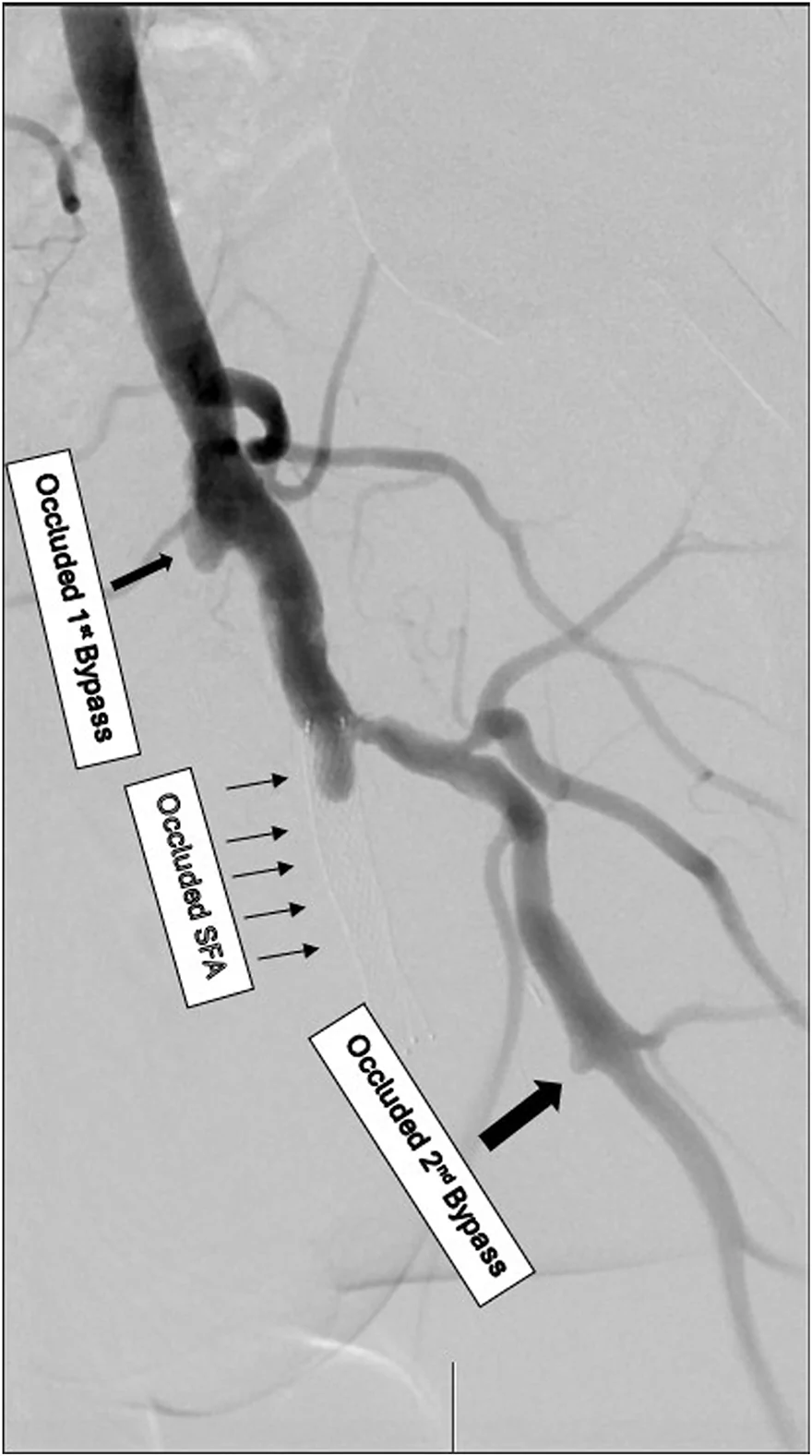
**Left groin angiography revealed chronically occluded Fem-Pop bypass, patent common femoral artery, totally occluded SFA, and occluded Profunda to tibial bypass.** SFA, superficial femoral artery.

**Figure 3:**
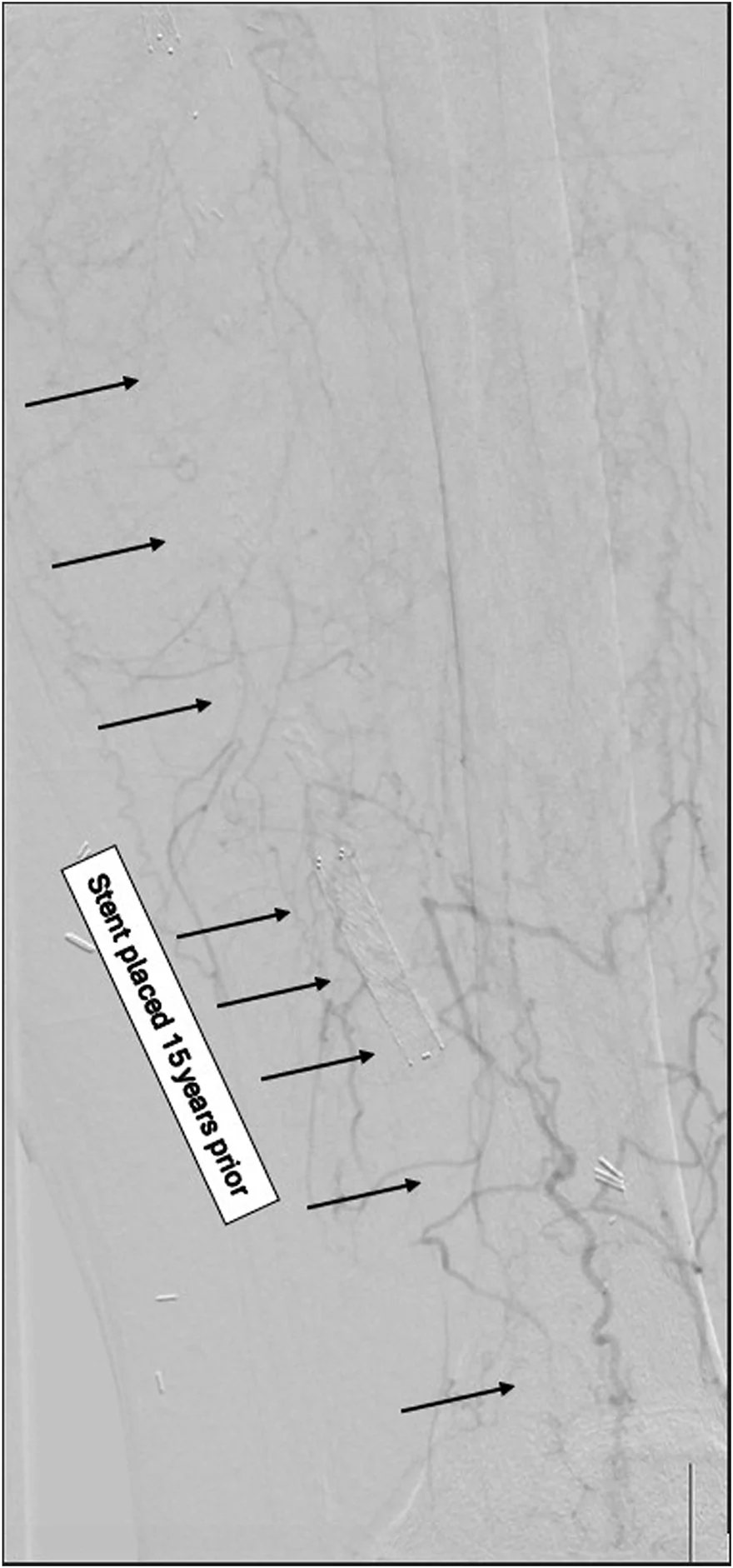
**Angiography of mid and distal SFA and popliteal arteries revealed totally occluded SFA and popliteal arteries.** A stent placed 15 years prior is visible in the distal SFA. SFA, superficial femoral artery.

His presentation with acute limb ischemia (Rutherford class 2A) in the setting of full anticoagulation with warfarin (INR 2.5) and DAPT was concerning. Of note, he had excellent risk factor control, with an LDL-C of 50 mg/dL, A1C of 6.2, and weight reduction on a GLP-1 agonist. An ankle-brachial index of the left leg showed monophasic flow in the left DP (ratio 0.35) and PT (ratio 0.34) arteries, with a toe-brachial index (TBI) of 0.00 (Figure 1). Due to a high index of suspicion and his personal history of recurring thromboses, he was taken for urgent peripheral angiography of the left leg via right common femoral artery access. Initiation angiography demonstrated complete occlusion of the left SFA, involving both proximal and distal stents, with flow reconstituting in the mid left AT artery via collateral filling from the left profunda femoral artery (Figures 2 and 3).

## Therapeutic intervention

**Figure 4:**
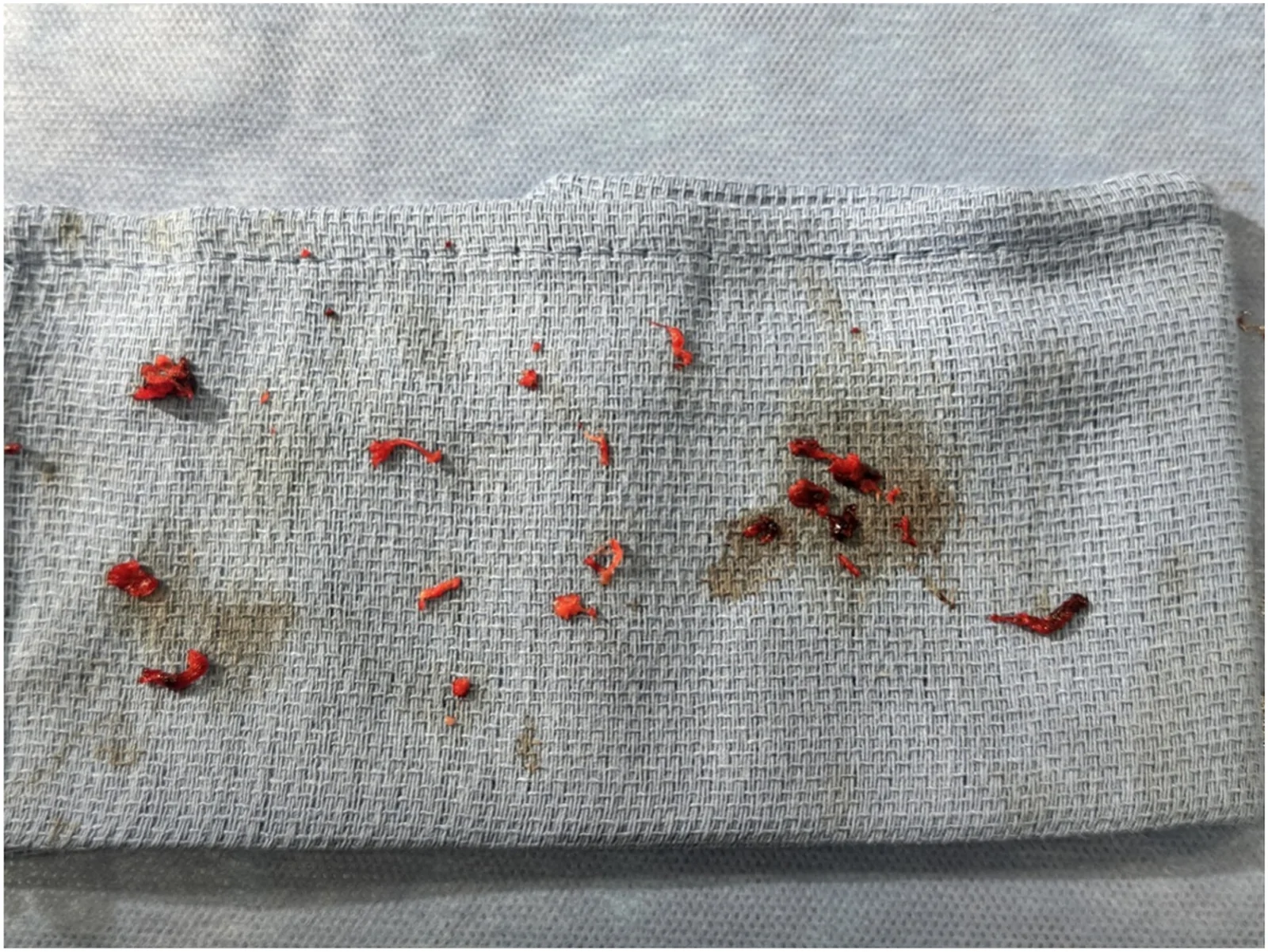
**Clot removedby mechanical thrombectomy.**

Proceeding with intervention, we delivered a 6F × 65-cm sheath from the right common femoral artery to the distal left external iliac artery. Advancing a 0.018-inch guidewire with a 0.018-inch support microcatheter to the proximal cap of the occlusion, the wire-microcatheter system crossed easily through the left SFA and thrombosed stents into the left peroneal artery. Several passes of mechanical thrombectomy were performed in the entire left SFA using the Pounce XL Thrombectomy System (Surmodics) (Figure 4). Upon retrieving the Pounce thrombectomy baskets, we noticed significant compression at the distal edge of the distal SFA stent, which was initially attributed to clot. However, despite multiple passes, the compression at this level did not improve.

**Figure 5:**
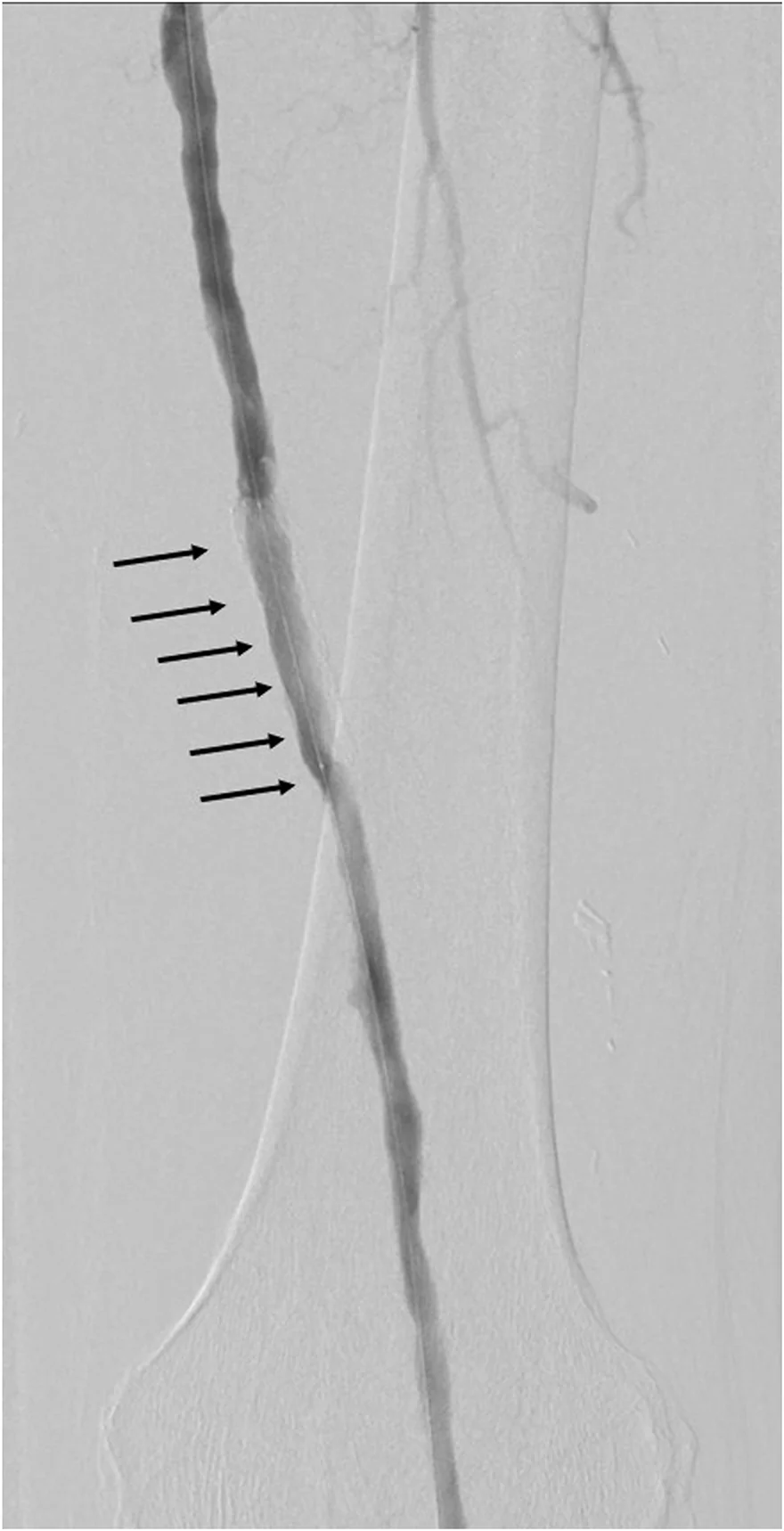
**Post-thrombectomy and PTA angiography, with normal flow but mild haziness starting at the proximal edge of the distal left SFA stent.** PTA, percutaneous transluminal angioplasty; SFA, superficial femoral artery.

Subsequent angiogram after thrombectomy showed partial recanalization of the left SFA and popliteal artery. We proceeded with further PTA of the entire left SFA and popliteal artery with sequential inflations of a 6.0 × 120 mm balloon, followed by PTA of the left AT artery with a 3.0 × 120 mm balloon to improve outflow. While this improved flow in the left SFA, the hazy appearance of the old stent in the distal left SFA suggested further disease (Figure 5).

**Figure 6:**
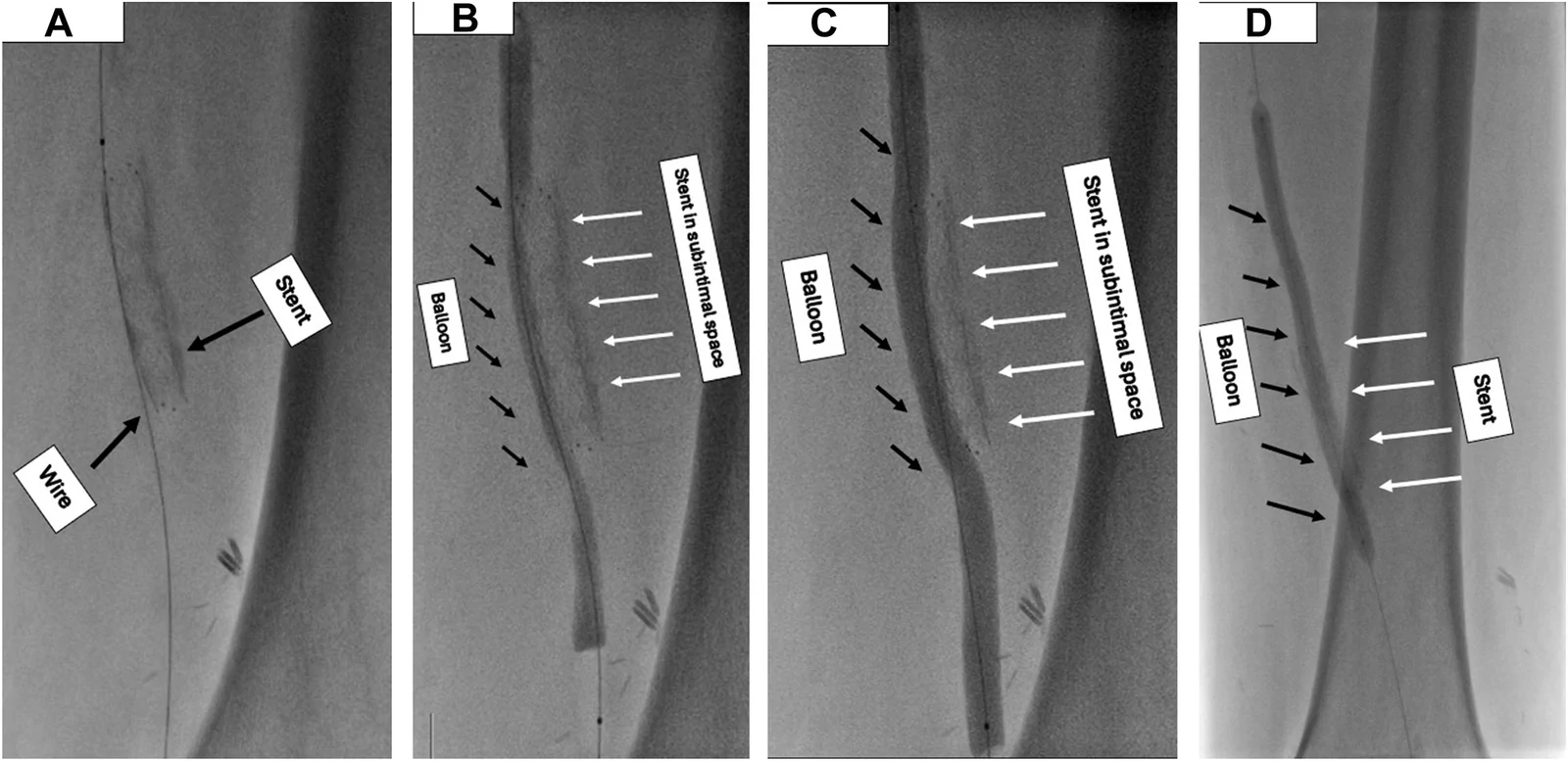
**“Sub-strut” wire in true lumen of SFA (A), followed by DCB angioplasty in the steep RAO view expanding the true lumen (compressed by the old subintimal stent) (B-C).** AP/LAO image failing to reveal the location of the stent relative to the balloon (**D**). RAO, right anterior oblique; SFA, superficial femoral artery.

**Figure 7:**
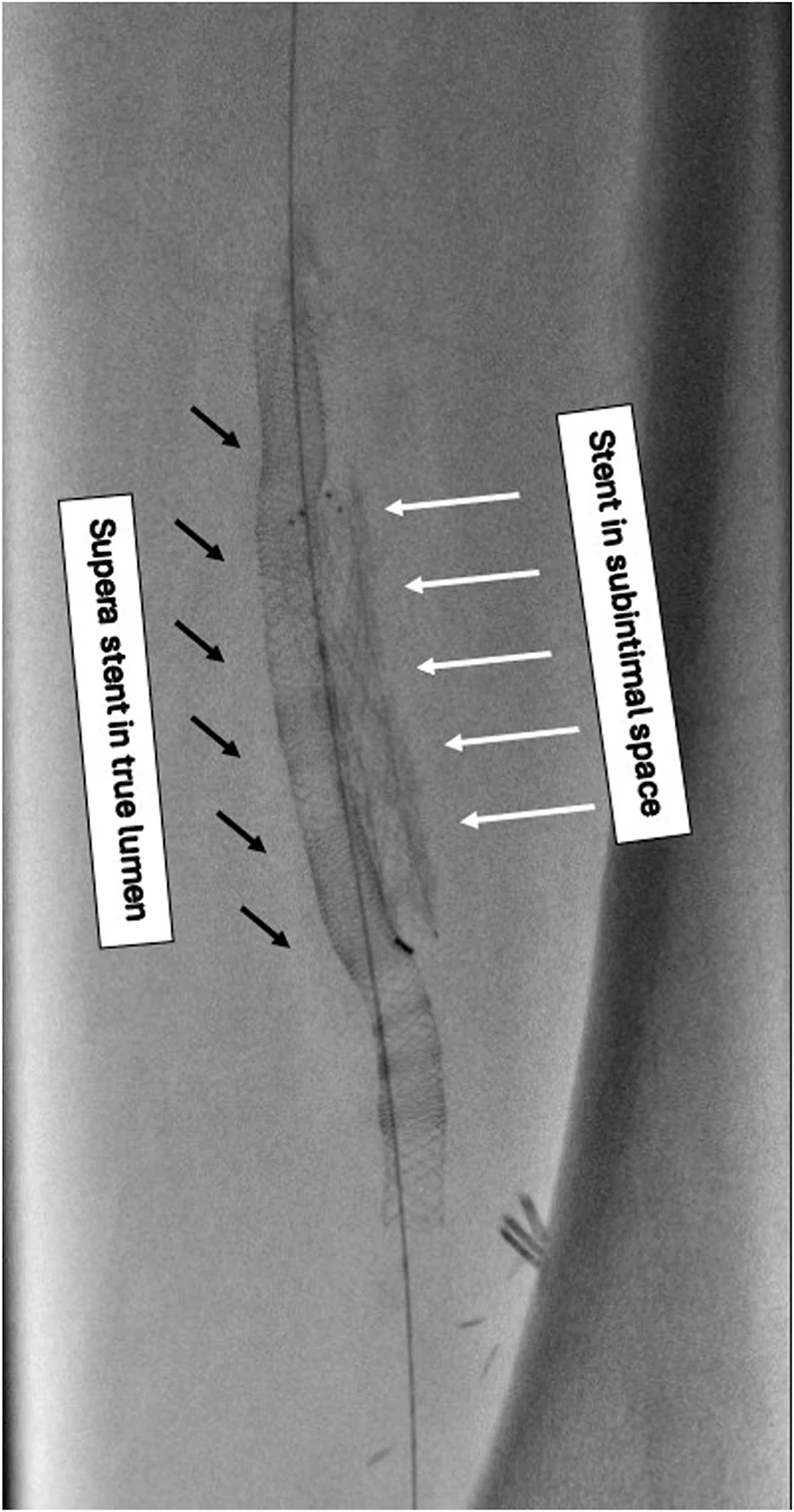
**Supera successfully implantedin true lumen, side-by-side to the subintimal stent.**

**Figure 8:**
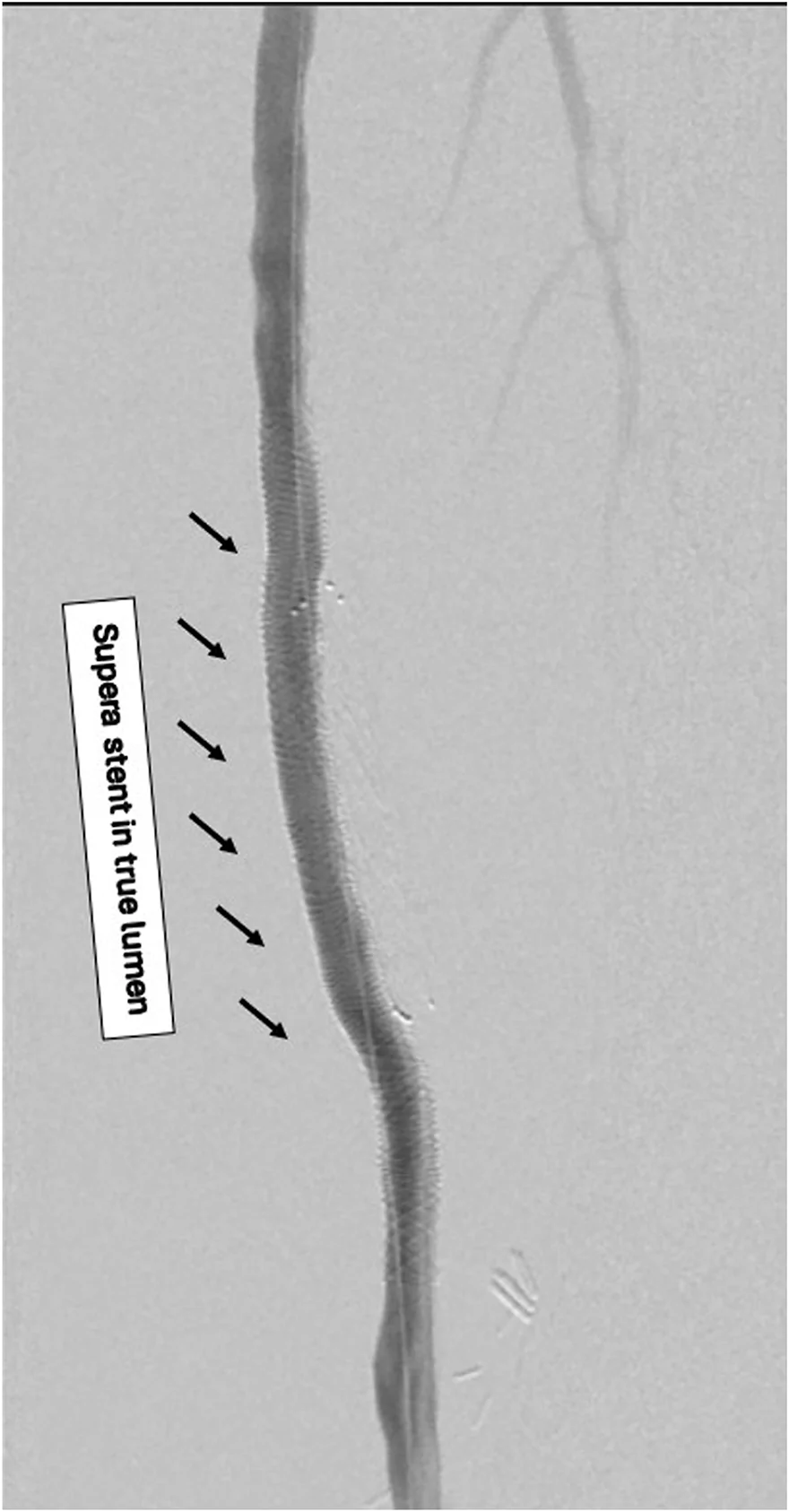
**Post-stent completion angiogram of distal left SFA.** SFA, superficial femoral artery.

Initially, it was felt the results were adequate given the presentation. However, minimal improvement was observed despite further angioplasty of the stent. The likelihood of being substrut or subintimal was also very low because the lesion was crossed very easily with 0.018-inch wire and the flow was reasonable. Additionally, multiple equipment passed through the stent without any challenges. Up to this point, all SFA and popliteal angiography and interventions were performed in the AP and LAO projections. Therefore, after taking a cine image of the wire and stent in the right anterior oblique (RAO) projections, we found our wire crossed the SFA independent of the stent (Figure 6A), indicating the stent was likely placed in the subintimal space 15 years ago and was the primary driver of recurring multiple SFA and popliteal occlusions in this patient. To confirm, several attempts were made to engage the stent in steep RAO views without success. This was further confirmed in the steep RAO projection while performing DCB angioplasty with a 6.0 × 120 mm balloon to expand the true lumen (Figure 6B). However, in the AP or LAO views, this finding was not visible at all (Figure 6C). To ensure durable patency of the native distal left SFA, a 5.5 × 120 mm Supera stent was deployed, carefully stacked in a side-by-side manner to ensure better radial strength (Figure 7). Final angiograms showed robust filling of the distal native SFA (Figure 8).

## Outcome and follow-up

**Figure 9:**
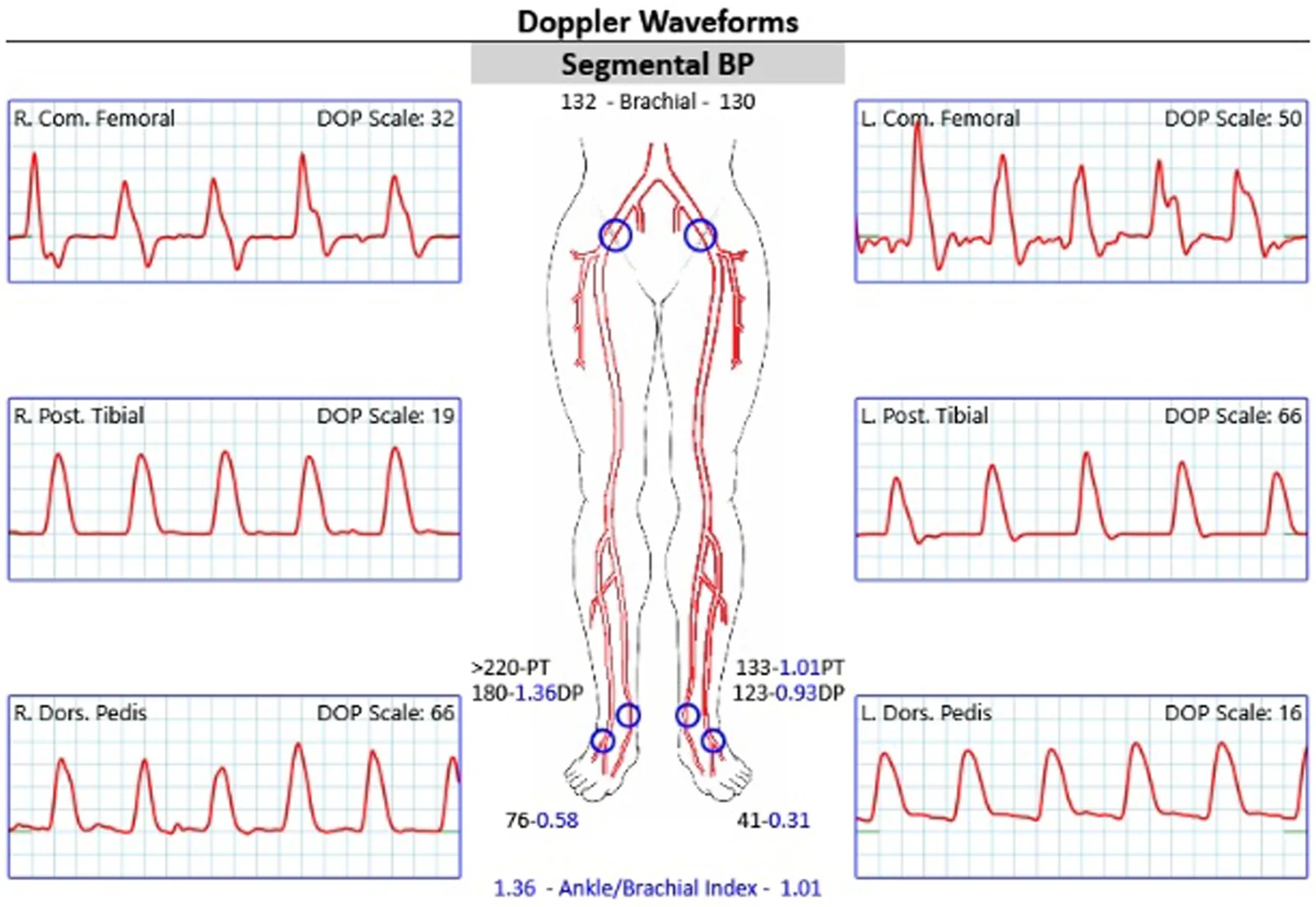
**Postintervention ABI showing biphasic flow in the left lower extremity.** ABI, ankle-brachial index.

The patient tolerated the procedure well without complications and reported a dramatic reduction in his left foot pain. After overnight observation, he was discharged home on DAPT and twice daily subcutaneous Lovenox injections. One month afterwards, he was seen in clinic, where his left foot was warm and without pain. Ankle-brachial index (ABI) showed biphasic flow in the left DP (ratio 0.93) and PT (ratio 1.01) arteries, with a TBI of 0.31 (Figure 9). He remains on DAPT and subcutaneous anticoagulation without any bleeding concerns.

## Discussion

This case presents a patient with an extensive history of left femoropopliteal revascularization who, despite adherence to a medication regimen of DAPT and oral anticoagulation, developed recurring thromboses and acute limb ischemia of the left leg. During our intervention, we discovered a prior stent in the distal left SFA deployed within the subintimal space, which had not only thrombosed but also effectively compressed the true lumen of the vessel, serving as the substrate for recurring thrombosis. Treating the true lumen by deploying a self-expanding stent in a “side-by-side” manner, we hoped to provide durable patency of the true lumen and reduce future obstructions at the distal left SFA level. We believe that prior interventions failed to distinguish the true lumen from the false lumen and led to revascularization that deceivingly appeared to recanalize the occluded stent. However, this likely only recanalized the true lumen without addressing the occluded subintimal stent or its compressive effects, thus creating the substrate for recurring thrombosis.

Secondly, the mechanism of stent failure in acute limb ischemia should be carefully evaluated. Stent thrombosis can present as acute limb ischemia, whereas in-stent restenosis typically presents gradually as claudication.^1^ Stent thrombosis can be classified as acute, subacute, late, or very late, with very late occurring more than a year afterwards.^2^ Technical factors such as stent underexpansion, malapposition, and fractures are the primary cause of femoral stent thrombosis.^3^ In our case, fluoroscopic evaluation of the distal left SFA stent did not clearly show stent fracture, underexpansion, or malapposition. While intravascular imaging would have provided more conclusive assessment, we were unable to wire into the occluded stent despite numerous attempts. In addition, stents in the distal SFA and popliteal arteries are subject to mechanical forces (hip/knee flexion, extension, shortening, and torsion) that can lead to stent deformation.^3^ Older peripheral stents also cannot accommodate the mechanical stress and demand of motion and are more prone to kinking and fracture. If stenting is required in this anatomic segment, we prefer to use the Supera self-expanding stent (Abbott Vascular), which contains an interwoven, braided nitinol design that affords both flexibility and high radial strength.

In the absence of technical and pharmacologic factors to explain stent thrombosis, our key discovery was identifying that the thrombosed stent was in a separate subintimal plane compressing the true lumen, and our interventions were treating the true lumen. Careful angiographic assessment following initial angioplasty and the use of different orthogonal projections helped to uncover this, as we followed the passage of wire and balloons relative to the old stent. This discovery allowed us to treat the external compression with a self-expanding stent, eliminating the recurring substrate of thrombosis. It is important to maintain a high index of suspicion when recurring vessel thrombosis occurs. Our case highlights the importance of recognizing external mechanical compression of the true lumen by a subintimal stent as a rare cause of recurring thrombosis and limb ischemia.

## CRediT authorship contribution statement

**Gordon Wong:** Writing – review & editing, Writing – original draft, Conceptualization. **Pedro S. Teixeira:** Writing – review & editing. **Omar Sheikh:** Writing – review & editing. **Mehdi H. Shishehbor:** Writing – review & editing, Visualization, Conceptualization.

## Declaration of competing interest

Mehdi Shishehbor is on the global advisory board for Abbott Vascular, Medtronic, Boston Scientific, ANT, Inquis Medical, and Inari. Gordon Wong, Pedro Teixeira, and Omar Sheikh do not have any disclosures.
